# Performance of Hybrid Photocatalytic-Ceramic Membrane System for the Treatment of Secondary Effluent

**DOI:** 10.3390/membranes7020020

**Published:** 2017-03-28

**Authors:** Lili Song, Bo Zhu, Stephen Gray, Mikel Duke, Shobha Muthukumaran

**Affiliations:** 1 Institute for Sustainability and Innovation, College of Engineering and Science, Victoria University, PO Box 14428, Melbourne, VIC 8001, Australia; songll1987@163.com (L.S.); Bo.Zhu@vu.edu.au (B.Z.); Stephen.Gray@vu.edu.au (S.G.); Mikel.Duke@vu.edu.au (M.D.); 2 College of Biological and Environmental Engineering, Zhejiang University of Technology, Hangzhou 310032, China

**Keywords:** ceramic membrane, photocatalysis, secondary effluent, fouling, size exclusion chromatography

## Abstract

Evaluation of an advanced wastewater treatment system that combines photocatalysis with ceramic membrane filtration for the treatment of secondary effluent was undertaken. The results showed that, after photocatalysis and ceramic membrane filtration, the removal of dissolved organic carbon and UV_254_ was 60% and 54%, respectively, at a concentration of 4 g/L of TiO_2_. Dissolved organic matter (DOM) present in the secondary effluent was characterised with a liquid chromatography-organic carbon detector (LC-OCD) technique. The results showed low removal of humics, building blocks, the other oxidation by-products and no removal of biopolymers after TiO_2_/UV photocatalytic treatment. This suggested that the radical non-selective oxidation mechanisms of TiO_2_/UV process resulted in secondary effluent in which all of the DOM fractions were present. However, the hybrid system was effective for removing biopolymers with the exception of low molecular weight (LMW) compounds acids, which accumulated from the beginning of the reaction. In addition, monitoring of the DOM fractions with LC-OCD analysis demonstrated that the reduction of the effluent aromaticity was not firmly correlated with the removal of humic substances for the combined processes.

## 1. Introduction

Recycled wastewater has been considered a reliable source for water supply in areas facing severe water shortages. However, secondary effluent from wastewater treatment plants (WWTPs) contains colloidal particles, pathogenic microorganisms and organic pollutants, as well as natural organic matter (NOM) that is not degraded in the biological treatment process. The presence of NOM can result in colour and odour, and can contain precursors for disinfection by-product formation increasing human health risks [1].

In order to increase the use of recycled water by communities, secondary effluent needs to undergo numerous treatment processes until a safe and reusable quality can be achieved. Over the last few decades, a great deal of interest has focused on advanced oxidation processes (AOPs) for the degradation of organic compounds present in wastewater. Among the AOPs, photocatalytic oxidation mediated by a semiconductor catalyst is one of the possible advanced oxidation processes. In particular, photocatalysis with TiO_2_ has been the focus of numerous investigations in recent years [2,3].

In the TiO_2_/UV photocatalytic oxidation process, hydroxyl radicals (·OH) are generated when catalyst (TiO_2_) is illuminated by ultraviolet (UV) light. As a result, organic compounds are mineralized to CO_2_, H_2_O and inorganic constituents without the use of chemicals, thus avoiding the need for purchasing of chemicals and the associated chemical waste problems [4,5]. In recent years, photocatalysis and membrane filtration have been proposed as a means for effective wastewater reclamation treatment [6]. This hybrid system combines the advantages of both membrane filtration and photocatalytic degradation of contaminants. Membrane filtration is not only able to separate suspended catalysts, but can also enhance the effluent quality by selective separation at the molecular level if small pore size memranes are used [6]. Furthermore, semiconductor photocatalysis is able to mineralize compounds causing membrane fouling and thus improve the consistency of the membrane’s operation [4,7,8].

Several studies have reported on the performance of the photocatalysis and membrane hybrid system for the removal of organic contaminants from water and wastewater. In particular, this hybrid process was used to improve membrane flux and minimise fouling, as well as recovery of the TiO_2_ catalyst [8,9]. For example, Lee et al. observed that there was a significant flux decline occurred when a mixture of humic acid (HA) and TiO_2_ without UV irradiation was applied during ultrafiltration (UF). This is because the formation of a dense filter cake with HA molecules deposited in the void space of the TiO_2_ particle layers. On the other hand, in the presence of UV light, no flux decline was observed during 6 h of experimentation. The authors explained the observed phenomenon in terms of decomposition of HA into non-aromatic by-products that reduced the extent of organic compound adsorption onto TiO_2_ particles compared to the initial HA compounds [9]. Lin et al. found that natural humic substances and other impurities in wastewater retard the photo degradation rates to a greater extent than commercial HA in the TiO_2_/UV system [10]. This is because the HA content of a water body can vary in composition in the natural environment, and thus the effects of natural humic substances cannot be accurately represented by a single commercial HA.

On the other hand, Le-Clech et al. considered the hybrid process for polishing treatment of surface water with a total organic carbon (TOC) concentration of 3 mg/L [11]. They found that the polyvinylidene fluoride membrane process removed around 18% of the initial TOC concentration, while adsorption on TiO_2_ of 0.1 g/L and UV light alone achieved 5% and 70% TOC removal, respectively. They also observed synergistic effects when all the three process were used together. Kim et al. used a TiO_2_ mediated photocatalytic membrane reactor using submerged microfiltration (MF) membranes for the treatment of lake water and seawater [12]. The authors observed that degradation of organic compounds in the seawater was minimal, whereas 80% removal of TOC was achieved in the case of lake water. The low mineralisation rate in the earlier case was mainly due to the presence of chloride ions in seawater, which were responsible for scavenging of the hydroxyl radicals, thus reducing the photo degradation efficiency. However, the authors did not find any significant membrane fouling during 4 h of filtration with seawater.

In their study, Ho et al. demonstrated that photo degradation with TiO_2_/UV could effectively reduce membrane fouling and enhance the permeate flux of a submerged polyethylene membrane reactor when treating biologically treated sewage effluent [6]. Similar conclusions were reported by Pidou et al. [13], who found that photocatalytic pre-treatment of synthetic grey water reduced membrane fouling when a minimum UV residence time of 120 min in the continuous stirred-tank reactor (CSTR) was used. All of these studies demonstrated that application of photocatalytic treatment improves permeate flux due to decomposition of organic molecules.

All of the previous studies exclusively focused on the use of polymeric membranes, while little research has been performed with ceramic membranes. The use of ceramic membrane as an alternative to polymeric membrane has gained interest in water and wastewater treatment due to their superior physical integrity, chemical resistance and thermal stability, and, in turn, has lower chemical demand, lower cleaning frequency and longer lifetime compared to their polymeric counterparts [14,15]. This suggests that ceramic membranes can operate at higher fluxes and tolerate extreme cleaning procedures without compromising membrane integrity. The decreasing cost of ceramic membranes, coupled with the recent successful development of others’ hybrid membrane processes such as ceramic-ozone process for wastewater applications [15], allow new perspectives for the ceramic membrane-TiO_2_/UV hybrid process. However, few studies have investigated the use of TiO_2_/UV photocatalytic treatment as a pre-treatment to ceramic membranes. For example, Benotti et al. used TiO_2_/UV and ceramic microfiltration membrane for the removal of pharmaceuticals, endocrine disrupting compounds and estrogenic activity from Colorado River water [16]. It was reported that twenty-nine of the targeted compounds, in addition to total estrogenic activity, were removed by more than 70%, while only three compounds were removed by less than 50% with the highest level of treatment. No estrogenically active transformation products were formed during the treatment. However, the authors did not present any data on permeate flux behaviour during the process, and given the different surface and adsorption properties of ceramic membranes compared to polymeric membranes, this remains a significant unresolved issue.

The performance of the photocatalytic process is influenced by the various compounds present in the secondary effluent. In particular, the presence of cations and anions in the secondary effluent causes part of the catalyst surface to become unavailable for photon absorption and organic matter adsorption, thus resulting in lower catalytic reaction [17]. Generally, the presence of various ions may affect the degradation rate via adsorption of the pollutants, reaction with hydroxyl radicals and absorption of UV light. There are several studies in the literature regarding the effects of various anions and cations [18,19,20]. These studies demonstrated that CO_3_^2−^, HCO^3−^ act as radical scavengers and also affect the adsorption process, and that Cl^−^ ions affect the adsorption step strongly, absorb UV light and have an undesirable effect on the photo degradation process, whereas other anions such as sulphate, phosphate and nitrate affect the degradation efficiency slightly. Yawalkar et al. have studied the effect of SO_4_^2−^, CO_3_^2−^, Cl^−^ and HCO^3−^ ions on the overall degradation rates of phenol solutions and reported that detrimental effects on photocatalytic oxidation were observed in the order SO_4_^2−^ < CO_3_^2−^ < Cl^−^ < HCO^3−^ [20]. Furthermore, anions such as Cl^−^, SO_4_^2−^, HCO^3−^, NO^3−^ and cations such as Na^+^, Ca^2+^ and Mg^2+^ present in the waters can be bound to TiO_2_ particles or very close to its surface, so that they can have substantial effects on the interfacial behaviour of the TiO_2_ particles [21].

Generally the presence of cations such as Na^+^, Ca^2+^, K^+^ and Mg^2+^ in the secondary effluent retards the photo degradation rates. These species are likely to reduce the rates of oxidation of organic compounds by competing for the oxidizing radicals or by blocking the active sites of the TiO_2_ catalyst [22]. According to Wang et al., cations such as Na^+^, K^+^, Ca^2+^ and Mg^2+^ are the common cations in natural water. They are all in the highest and most stable oxidation state and cannot capture electrons in the solution. It is hypothesized that these metal ions would not have a significant impact on the photodegradation [23]. While it has been reported that these simple inorganic cations showed a slight suppression of the degradation reaction, this could be the effect of Cl^−^ co-present in solution [22,23]. This is because Cl^−^ ions might inhibit the photodegradation due to adsorption on the surface of photocatalyst. It is also suggested that there is competitive adsorption by anions such as bicarbonate, sulphate, chloride and organic matter on the surface of catalyst, especially at lower concentrations of organic matter. Therefore, wastewater composition remains an important parameter in the overall performance of the photocatalysis.

Our previous work described the influence of experimental variables such as solution pH, salinity and TiO_2_ dose on the removal of a model HA solution using TiO_2_/UV photocatalytic oxidation process and ceramic membrane filtration [8,24]. These studies showed that relatively high removal of TOC and UV absorbance, removal of HA fouling potential and the complete recovery of TiO_2_ slurry using this hybrid system were achieved. Furthermore, it was shown that the presence of salt reduces the surface contact between the pollutant and the photocatalyst, and thus reduced photo degradation efficiency. However, for practical application, the feasibility of the combined photocatalysis and ceramic membrane filtration for the treatment of wastewater rather than model HA solution is of more interest. This is because membrane fouling varies with the characteristics of organic compounds present in wastewater, as does the oxidation efficiency of organic compound degradation by TiO_2_/UV.

Therefore, laboratory studies considered the efficiency of TiO_2_/UV with ceramic membranes for the treatment of a disinfected secondary effluent. The secondary effluent contained dissolved organic carbon (DOC) of approximately 9–10 mg/L, and a liquid chromatography-organic carbon detector (LC-OCD) technique was used to characterise the organic compounds in the effluent. To our knowledge, the present study is the first to report the application of this hybrid system to treat secondary effluent, and to characterise the organic compounds present at various stages of this hybrid process with LC-OCD analysis.

## 2. Materials and Methods

### 2.1. Materials

UV and chlorine disinfected secondary effluent was collected from the Western Treatment Plant (WTP) in Melbourne. Other characteristics of the secondary effluent are presented in Table 1.

Titanium dioxide P25 (Evonik, Essen, Germany, 80% anatase, 20% rutile, 99.8% purity, average particle size 30 nm and specific surface area of 50 m^2^/g) was used as the photocatalyst. The TiO_2_ catalyst was obtained as a dry powder and stored at room temperature. The desired amount of TiO_2_ powder was weighed and mixed with a small quantity of deionized water (electric resistivity: 10–15 MΩ·cm, TOC: < 30 µg/L and pH: 6.5–7.5) to prepare TiO_2_ slurry prior to each set of experiments. For this system, the particle size of the TiO_2_ (P25) after dispersion with deionized water was measured as 300 nm, suggesting the TiO_2_ particles were agglomerated in suspension. Analytical grade chemicals were used without further purification in all experiments.

### 2.2. Apparatus

**Figure 1:**
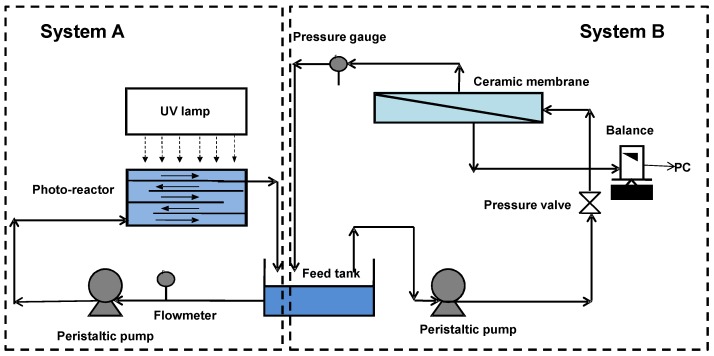
Schematic of a laboratory-scale photocatalysis (System A)/ceramic membrane system (System B).

The hybrid set up shown in Figure 1 consisted of a batch photocatalytic reactor (System A) and cross flow ceramic membrane unit (System B). The photocatalytic reactor was made of stainless steel with baffle plates, such that the water flowed in a zigzag manner through 5 open channels. The volume of the photocatalytic reactor was 2 L and the total illuminated surface area of the reactor was 713 cm^2^. The UV panel consisted of six 18W UV-A lamps (NEC Blacklight Lamp, Tokyo, Japan). UV-A intensity was measured by a UV irradiance meter with a range of 320–400 nm (UV-A, Photoelectric Instrument Factory of Beijing Normal University, Beijing, China). The tubular titania ceramic ultrafiltration membrane (Schumasiv^TM^, Pall Co., Australia) made of a titania (TiO_2_) coating on an alumina (α-Al_2_O_3_) support with pore size of 5 nm (approximate molecular weight cut off of 10 KDa) and an effective surface area of 48.38 cm^2^ was used. This membrane was chosen for its relative hydrophilicity and the ability to reject the TiO_2_ slurry particles given its small pore size [24].

### 2.3. Experimental Procedures

Disinfected secondary effluent from the one of Melbourne’s Wastewater Treatment Plants (WWTPs) was used for all of the experiments. Before each photocatalytic treatment, TiO_2_ slurry and secondary effluent were mixed by a magnetic stirrer in the dark for 20 min in order to obtain adsorption equilibrium. The solution (2 L) to be treated was pumped from the feed tank into the photocatalytic reactor at a flow rate of 0.5 L/min and was irradiated by UV for 1 h and at a UV intensity of 3.4 mW/cm^2^. Based on the UV irradiation time and hydraulic retention time of the suspension in the reactor, the average exposure time to UV irradiation was 20 min. All of the experiments were carried out at 20 °C.

Ceramic ultrafiltration experiments were carried out at a constant transmembrane pressure (TMP) of 100 kPa that varied by less than 5 kPa throughout the filtration process. The treated slurry from the batch photocatalytic reactor was fed into the ceramic membrane unit for further treatment. The flux was continuously measured using a balance that recorded the filtrate weight throughout filtration via data acquisition software. All filtration experiments recycled both the retentate and filtrate back to the feed tank. The experiment was carried out to evaluate the individual effect of photocatalysis and ceramic membrane treatment of secondary effluent. Secondly, the influence of the hybrid system on the treatment of secondary effluent was evaluated. Most of the experiments were carried out in duplicate. The clean water flux varied between experiments was 22.8 ± 0.3 L·m^−2^·h^−1^. The permeability of the 5 nm membrane was found to be 0.29 ± 0.002 L·m^−2^·h^−1^·kPa^−1^.

### 2.4. Analyses

#### 2.4.1. DOC and UV_254_ Measurements

The performance of the hybrid system was monitored through two main output parameters: DOC and UV 254 nm absorbance removal. Over the duration of the experiment, 50 mL samples were collected at designated time intervals and filtered through 0.45 μm membrane filters. The DOC concentration of the samples was measured using a Shimadzu TOC V-CSH analyser (Tokyo, Japan). The presence of aromatic organic constituents in the water sample was indicated by measuring the absorption of the filtered sample at a wavelength of 254 nm against organic-free water as blank (UV_254_-UV absorbing, Method 10054, HACH, DR 5000 spectrophotometer, Loveland, CO, USA). Within the duration of the experiments, the quality of the secondary effluent varied only slightly with an average DOC concentration of 9.5 ± 0.5 mg/L and the UV absorbance of 0.16 ± 0.01 cm^−1^.

#### 2.4.2. LC-OCD Analysis

Apparent molecular weight distributions (AMWD) of dissolved organic matter in the samples were determined by liquid chromatography (LC) coupled with UV_254_ detection (UVD), organic carbon detection (OCD) and organic nitrogen detection (OND). This technique is considered a reliable method for characterising organic matter in wastewater samples since the technique quantifies biopolymers, humic substances and low molecular weight (LMW) organic fractions (including LMW acids and neutrals). The molecular size distribution was measured and correlated to molecular weight using LC-OCD and OND system model 8, based on the Gräntzel thin film reactor developed by DOC Labor, Dr. Huber, Karlsruhe, Germany, using a Toyopearl HW-50S weak cation exchange gel filtration column (Sigma-Aldrich, St. Louis, MO, USA). The fractions coming from the column were measured by UVD followed by OND and OCD. The International Humic Substances Society (IHSS) Suwanee river reference material, HA and fulvic acid (FA) were used as reference samples. The chromatograms obtained were interpreted using DOC-Labor ChromCALC software (Karlsruhe, Germany). The organic carbon detector is capable of detecting the presence of all organic compounds, making it reliable for all waters. An approximation of protein and polysaccharide concentrations can be made based on the organic carbon and nitrogen concentrations from the organic carbon and organic nitrogen detectors for the biopolymer fraction. The chromatogram results are reported against retention time in minutes. Analysis (DOC-Labor) was carried out by TU Dresden in Germany.

## 3. Results and Discussion

### 3.1. Effect of TiO_2_ Concentration on Photocatalytic Treatment

**Figure 2:**
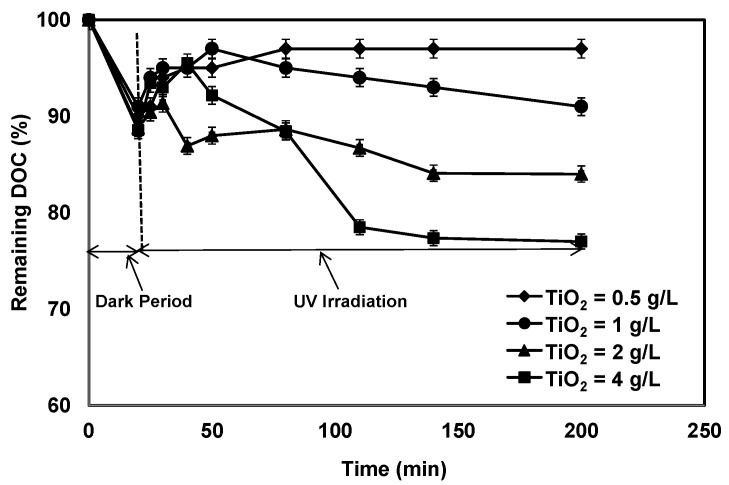
The remaining DOC as a function of irradiation time at different TiO_2_ concentrations (UV intensity = 3.4 mW/cm^2^; pH: 7.5).

Figure 2 shows the DOC removal over time at various TiO_2_ concentrations. Experiments were conducted for 200 min using various TiO_2_ concentrations (0.5, 1, 2 and 4 g/L) to evaluate the degradation rate of organic matter in secondary effluent. The initial DOC concentration of the secondary effluent was 9.5 ± 0.5 mg/L. In Figure 2, the first point at 20 min was measured without UV (dark period), while the remaining points were measured in the presence of UV. The first 20 min of the reaction shows the adsorption of organic matter on the TiO_2_ surface in the dark, ranging from 9% to 12% DOC removal between 0.5 and 4 g/L of TiO_2_. This result shows that there was no significant difference in the adsorption even at high TiO_2_ concentration. This suggested that only a portion of organics could adsorb on the TiO_2_ surface. This is consistent with the study by Le-Clech et al., where they reported that the amount of NOM removal due to adsorption on the surface of this photocatalyst powder was 5% [11]. After 20 min the UV exposure commenced, and a slight increase in DOC concentration was observed prior to continued decrease in DOC for all TiO_2_ concentrations except at low concentration (0.5 g/L). The increase in DOC was explained by the release of intermediate UV oxidation products from the TiO_2_. These intermediate products are more hydrophilic than the organic matter that was originally present in the secondary effluent as a result of oxidation and do not adsorb as strongly on the TiO_2_ surface, thus dissolving back into the bulk water [25]. Subsequent decrease in DOC measured was attributed to mineralization of intermediate products due to photocatalytic oxidation. Within the 200 min of reaction time (20 min in dark and 180 min with UV exposure), 3%, 9%, 16% and 23% DOC removal was achieved with TiO_2_ concentrations of 0.5, 1, 2 and 4 g/L, respectively. This result suggests that the TiO_2_ catalyzed photo-oxidation system selectively favours species that are easily degraded [10]. The results show that all of the organic matter in the secondary effluent was not effectively removed by the photocatalytic treatment even at higher TiO_2_ concentrations, even though availability of active sites increased for the higher TiO_2_ concentrations. Generally, with an increase of TiO_2_ concentration, the surface area of the catalyst increases; therefore, the adsorption capacity and degradation efficiency would increase. Alternatively, the elevation of photocatalyst concentration enhances the opacity of suspension and decreases the diffusion of photon flux in the suspension. In addition, agglomeration of TiO_2_ particles may reduce the effective total surface area of the photocatalyst [26]. Therefore, above a certain TiO_2_ concentration, the adsorption rate or pollutants degradation rate remains constant and even decreases with increasing TiO_2_ concentration.

**Figure 3:**
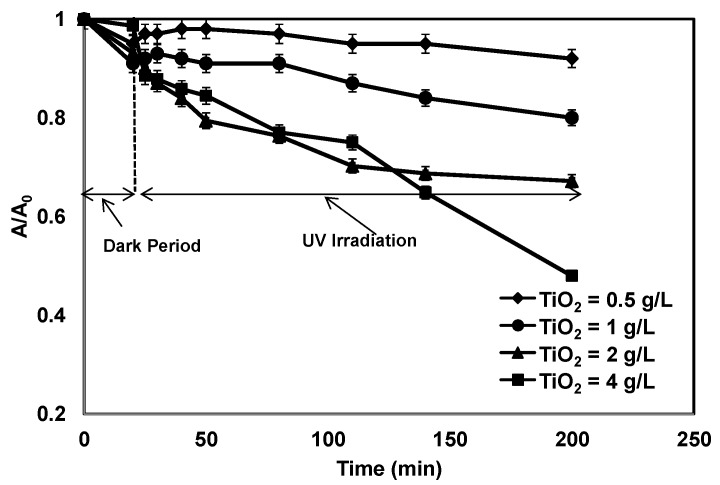
The reduction in UV absorbance as a function of irradiation time at different TiO_2_ concentrations (UV intensity = 3.4 mW/cm^2^; pH: 7.5).

Figure 3 represents the relative UV absorbance at 254 nm for the water samples obtained from the tests at different TiO_2_ concentrations. The overall reduction in UV absorbance after 200 min of reaction time (20 min dark and 180 min with UV exposure) was 8%, 20%, 33% and 52% for TiO_2_ concentrations of 0.5. 1.0, 2.0 and 4.0 g/L, respectively, which was twice that of the corresponding DOC removal. Since the UV_254_ absorbance is a surrogate parameter to assess the content of aromatic moieties, the above results revealed that the organic compounds were partially oxidized by photocatalysis, but were not completely mineralized. A control tests with model HA in pure water was conducted with the same photocatalysis-ceramic membrane hybrid system under the same hydraulic and photocatalytic operating conditions (results not shown). Substantial removals (>80%) of DOC and (>90%) UV_254_ absorbance was achieved using 0.5 g/L of TiO_2_ and within 120 min of reaction time, which was considerably different from the results of Figure 2 and Figure 3.

Generally, secondary effluent contains biological treatment by-products, dissolved organic and inorganic compounds mixed together with a smaller concentration of toxic pollutants. Competition effects for a mixture of compounds are important because if the TiO_2_ catalyzed photo-oxidation system reacts preferentially with certain compounds, its use for treating less competitive compounds will be inefficient. Such effects for organics in this wastewater are evidenced by the LC-OCD analysis, which is discussed in the later section.

The specific UV absorbance (SUVA), which is defined as SUVA = (UV_254_/DOC) × 100, was determined with and without TiO_2_ photocatalytic oxidation using different TiO_2_ concentrations for the treated wastewater. SUVA represents the proportion of total organic compounds present that contain unsaturated carbon bonds and aromatic groups, as these functional groups strongly absorb at 254 nm. Natural waters with high SUVA values have values greater than 4 L/m·mg, and are composed of relatively high contents of hydrophobic, aromatic and high MW NOM fractions, while waters with SUVA at less than 2–3 L/m·mg contain mostly hydrophilic, non-humic and low MW fractions [27]. The SUVA value for the wastewater feed was 1.7 ± 0.1 L/m·mg, which is low compared to other waters and indicates a low proportion of aromatic compounds within the total organic compounds present. Following the 200 min photocatalytic treatment, the SUVA_254_ values of the treated effluent varied from 1.26, 1.24, 1.11 and 0.73 L/m·mg with TiO_2_ concentrations of 0.5, 1, 2 and 4 g/L, respectively. The SUVA values of the treated effluent decreased in the same order as observed for UV_254_, demonstrating that the organic compounds were transformed to less aromatic configurations and that higher-SUVA components of the organic matter were preferentially removed from solution. However, the overall SUVA removal efficiency was less than 60% even at 4 g/L TiO_2_ concentration compared to model systems containing HA, where the SUVA removal efficiency was greater than 95% after 200 min photocatalytic oxidation using 0.5 g/L TiO_2_ (data not shown). Two possible reasons for the lower efficiency of SUVA removal was observed for the wastewater compared to the model water are proposed: (1) the chemical structure of organic molecules present in the secondary effluent is different from the model HA and are less amenable to photocatalysis; and (2) secondary effluent contains various salts that may interfere with photocatalysis.

### 3.2. Effects of TiO_2_ Slurry Concentration on Membrane Flux and DOC and UV_254_ Removal

**Figure 4:**
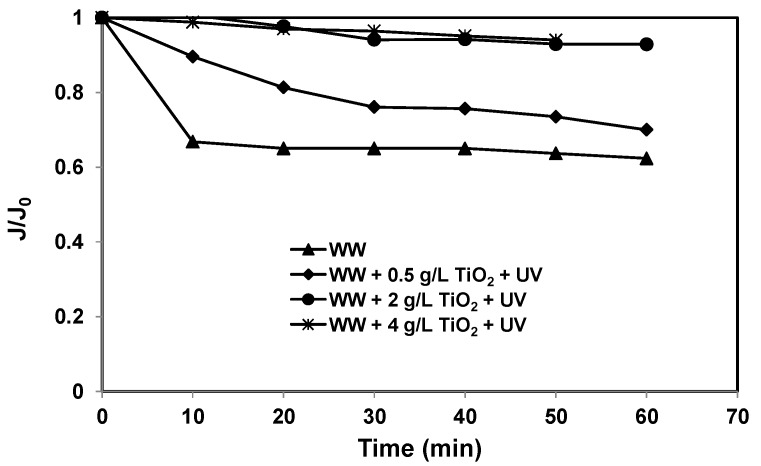
Permeate flux of the photocatalytic—ceramic membrane hybrid system for various TiO_2_ concentrations (TMP: 100 kPa, cross-flow velocity (CFV): 0.4 m/s, UV intensity: 3.4 mW/cm^2^; pH: 7.5).

Ceramic ultrafiltration membrane flux after photocatalysis with different TiO_2_ slurry concentrations (0.5, 2 and 4 g/L) was measured and compared with the flux without TiO_2_ slurry. The results are shown in the form of normalised flux, J/J_0_, in Figure 4. A slight flux decline was observed when 2 and 4 g/L of TiO_2_ slurry was used, but the flux decline was significantly greater at 0.5 g/L TiO_2_ and without TiO_2_ (30% and 40% with 0.5 g/L and 0 g/L TiO_2_, respectively, after 1 h operation). Generally, the permeate flux declined quickly within the first 30 min, following which the rate of decline was slow and became almost constant at longer filtration times. In this case of constant concentration (permeate and concentrate returned to the feed), a possible explanation is the build of the filtration cake, establishing rapidly at the beginning of the experiments, and then reaching an equilibrium. The formation of filtration cake on a membrane surface was formed by the deposition of both TiO_2_ particles (particulate matter) and contaminants (dissolved organic compounds) present in the secondary effluent. It can be postulated that the flux improvement with the use of higher TiO_2_ concentrations after photocatalytic treatment was attributed to the formation of a more porous cake layer with coarser surface as well as improved hydrophilicity of the membrane covered with TiO_2_ particles compared with lower TiO_2_ concentration (0.5 g/L). Thus, the degradation of organic matter present in the suspension or bound to the TiO_2_ particle surface must play a key role in the improvement of flux when UV irradiation was provided. On the other hand, higher flux decline during the treatment of secondary effluent alone was mainly due to the formation of a uniform cake layer on the membrane surface as well as fouling caused by clogging of pores or adsorption within the pores by dissolved matter.

After photocatalysis (20 min in dark and 60 min with UV exposure), the DOC removal was 3%, 16% and 23% and the UV_254_ removal was 2%, 33% and 52% for 0.5, 2 and 4 g/L of TiO_2_ concentrations, respectively (Table 2). However, after the hybrid system (photocatalysis and ceramic filtration of 60 min), the DOC removal was 18%, 36% and 60%, and the UV_254_ removal was 10%, 52% and 54% for 0.5, 2 and 4 g/L of TiO_2_ concentrations, respectively (Table 2). Negligible flux decline and slightly higher DOC and UV_254_ removal was observed when 4 g/L of TiO_2_ was used. In our previous work, we have observed that when HA was used as feed, the addition of TiO_2_/UV treatment did not affect the permeate flux through the ceramic membrane regardless of photocatalyst loading [24]. Furthermore, we found 70% and 96% of TOC and UV absorbance removal, respectively, after hybrid treatment (60 min photocatalysis with 0.5 g/L of TiO_2_ and 60 min ceramic membrane filtration). In that case, however, the reduction in TOC and UV absorbance removal and flux decline was observed when NaCl was present. This is because the presence of salts leads to agglomeration of TiO_2_ particles by suppressing the stabilising effects of electrostatic repulsion, and thereby reduces the effective surface contact between the pollutant and the photocatalyst, and particles within agglomerates are shielded from the UV radiation exposure. The results obtained in the present research, when secondary effluent was used as a feed, are quite different. Considering the composition of secondary effluent (Table 1), it can be concluded that the presence of various salts present in the secondary effluent affected the performance of both the photocatalysis and ceramic membrane filtration. In the presence of organic matter, TiO_2_ formed larger diameter clusters, thereby decreasing the resistance of the TiO_2_ cake layer formed on the membrane surface. The agglomeration size of the TiO_2_ particles varies as a function of its zeta potential and thus as a function of pH and electrolyte concentration. According to Le-Clech et al., the organic matter in the water can be removed either by adsorption on TiO_2_ particles or photo oxidation of organic matter due to the TiO_2_ in the presence of UV light [11]. In this study, there is little difference in the extent of organic matter adsorption on TiO_2_ surfaces at various TiO_2_ slurry concentrations as explained in the earlier section. However, there was a significant difference in DOC removal during the photocatalysis by TiO_2_/UV at various TiO_2_ concentrations_._

### 3.3. LC-OCD Analysis

**Figure 5:**
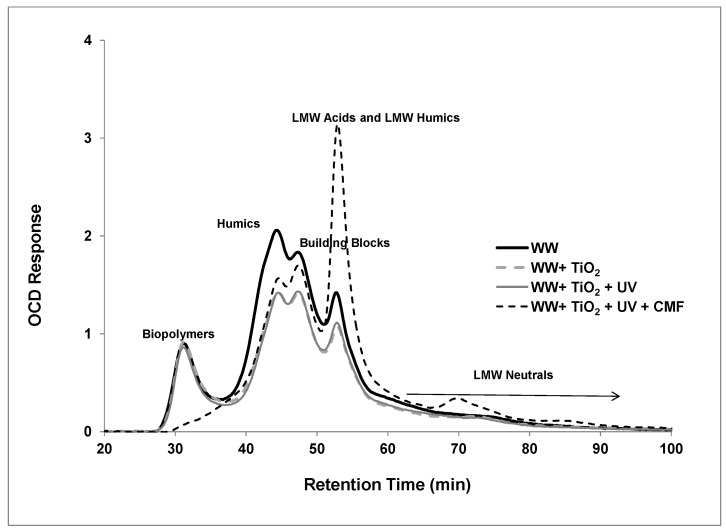
LC-OCD chromatograms of secondary effluent at various treatments as a function of retention time (TiO_2_: 0.5 g/L, TMP: 100 kPa, CFV: 0.4 m/s, UV intensity: 3.4 mW/cm^2^; pH: 7.5).

The chromatograms obtained with OC detection are shown in Figure 5. This technique allows the separation and assignment into different groups of organic components, particularly biopolymers (proteins, protein-like substances, polysaccharides), humic substances, building blocks (sub units of humic substances), low molecular weight acids (monoprotic organic acids) and low molecular weight neutrals (alcohols, aldehydes, ketones, amino acids). Figure 5 shows the evolution of LC-OCD chromatograms following different treatment as a function of retention time. These chromatograms provide better understanding of the effects of TiO_2_ adsorption without UV, the photocatalytic effect of TiO_2_/UV, and the hybrid system of photocatalysis and ceramic membrane filtration (CMF) on the secondary effluent. The first peak appeared at 29 min after sample injection and was assigned to the biopolymers with an approximate molecular weight (MW) greater than 20,000 Da. This was followed by humic substances with an MW of 1000–20,000 Da, building blocks with a MW of 300–500 Da, and low molecular weight acids of less than 350 Da at approximately 43, 46, and 52 min, respectively. The low molecular weight neutrals were detected at the end of the chromatogram between 55–100 min.

It can be seen from the LC-OCD analysis that humic substances and biopolymers represented the predominant fractions of organic carbon content in the secondary wastewater. After hybrid treatment, the LC-OCD chromatogram significantly changed in organic compound composition. After the dark period of TiO_2_/UV photocatalytic treatment (WW + TiO_2_), the removal of humics and building blocks present in the secondary effluent was observed, but the biopolymer peak did not change. This is reflective of greater adsorption of humics and building blocks on the surface of the TiO_2_ compared to negligible adsorption of biopolymers. Following 60 min UV treatment, there is no further removal of any class of organic components including humics and building blocks. As explained previously, the presence of high alkalinity and ions in the secondary effluent would minimise the photo degradation process due to the scavenging effects. In our previous study, we have shown that the TiO_2_/UV treatment was more efficient for treating water containing HA alone, but, in the presence of NaCl, the degradation efficiency decreased slightly [24]. Whereas in this study, secondary effluent contained various dissolved and colloidal organic compounds as well as various salts. The main difference between the secondary effluent sample and the sample after photocatalytic oxidation is the reduction in the amount of humic substances via hydroxyl radical oxidation. However, feed samples exhibited a clearly detectable biopolymer peak, and the biopolymer peak remained after TiO_2_ adsorption without UV and following photocatalysis with TiO_2_/UV, as there was no reduction of biopolymers detected by OCD. After TiO_2_/UV photocatalytic and ceramic membrane filtration (5 nm ceramic filtration), there was a complete removal of biopolymers, marginal removal of humics and an increase in LMW compounds. The result suggests that the increase in biodegradable materials such as LMW compounds may be either present in the secondary effluent or produced as a product of HS oxidation, which can easily pass through the 5 nm ceramic membrane [28]. According to Liu et al., these compounds are relatively inert towards chlorination and therefore produce low yields of disinfection by products [29]. The results also support biopolymers retained by the ceramic membranes contributing to membrane fouling. This is consistent with the study by Laabs et al., who found that the polysaccharide-like materials contribute significant fouling in UF membrane filtration of WWTP effluent [30]. Furthermore, Laabs et al. demonstrated in their study that the retention of polysaccharide-like materials led to gel type of fouling when the UF membrane was used to process wastewater effluent organic matter (EfOM) [30]. The biopolymer peak encompasses materials from 50,000 to >200,000 Da, identifying these compounds as being in the range of >5 nm. Therefore, the flux decline of ceramic membrane with a pore size of 5 nm is explained by the presence of the biopolymers. Hence, the hybrid system operated through hydroxyl radical oxidation and molecular separation, which progressively led to an effluent with lower biopolymer content and an increase of the LMW compounds. However, substantial amounts of humic substances still remained after hybrid treatment.

**Figure 6:**
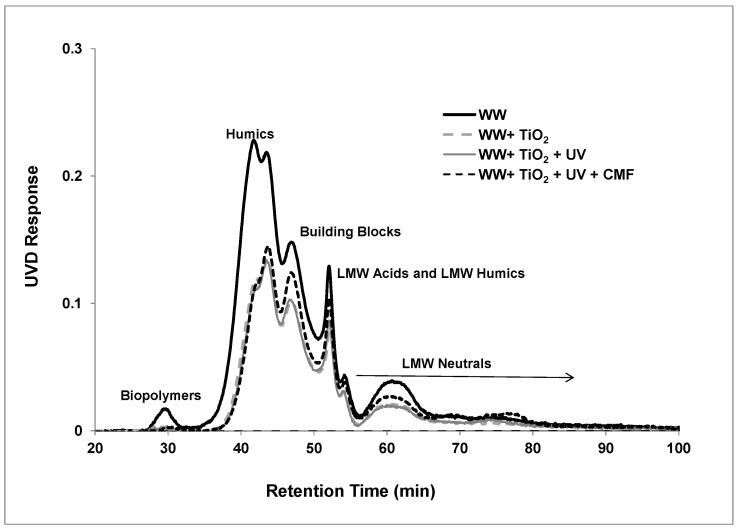
LC-UVD chromatograms of secondary effluent at various treatments as a function of retention time (TiO_2_: 0.5 g/L, TMP: 100 kPa, CFV: 0.4 m/s, UV intensity: 3.4 mW/cm^2^; pH: 7.5).

The corresponding UV_254_ chromatogram (Figure 6) shows a distribution similar to the organic carbon chromatograms. However, polysaccharides that are a significant component of the biopolymers cannot absorb UV light with a wavelength of 254 nm, as these compounds do not have the necessary C=C double bonds. Therefore, the biopolymer peak is reduced in comparison to the other organic compounds because the polysaccharides are not detected by UV_254_. However, a small peak in the biopolymer is detectable, which provides evidence of other substances besides polysaccharides eluting in the biopolymer peak, such as proteins or organic colloids that contain both the polysaccharides and proteins. This is consistent with the study by Laabs et al., where they have shown that organic colloids would elute in the biopolymers’ peak [30].

The SUVA parameter, which reflects the normalised aromaticity of waters, decreased from 1.8 L/m·mg to 1.37 L/m·mg and 1.33 L/m·mg after TiO_2_ adsorption and TiO_2_/UV photocatalytic treatment. This result indicates that the reduction of humic content is mainly due to the TiO_2_ adsorption. Because of the competition occurring between the different species, an expected decrease of photocatalytic performance was detected when humic substance was present along with the other specific pollutants [11]. The slight decrease in SUVA after TiO_2_/UV photocatalytic oxidation treatment could be explained by the reactions of humic substances with hydroxyl radicals to produce various hydroxylation products via oxidation of aromatic rings [28]. A similar result was observed by Wang et al., where they observed the formation of various hydroxylation products of aromatic carbons such as methylcatechols and methylhydroquinone during photocatalytic oxidation studies of cresols (commonly used to simulate NOM and humic substances) [31]. As seen from Figure 6, after photocatalytic treatment, the humic contents were still present in significant quantities and composed the major organic fraction in the effluent. The photocatalytic treatment provided enough oxidation of the humic substances to achieve high aromaticity depletion, but was not able to create adequate cleavage to achieve characteristic structure loss. This implies that aromaticity of the functional groups would be destroyed, but simpler substances would not be generated immediately. Therefore, the main structure would become smaller but remain unbroken. Due to the hybrid system, the SUVA parameter reduced to 1.18 L/m·mg. The ceramic membrane retained some of the humic substances after TiO_2_/UV photocatalytic oxidation treated effluent, and the smaller MW compounds that preferentially passed into the permeate had lower degrees of aromaticity, resulting in a slight decrease in the SUVA value.

## 4. Conclusions

The performance of the hybrid photocatalysis-ceramic membrane system for treatment of secondary effluent was investigated and discussed. The obtained results led to the following conclusions.

The DOC and UV_254_ removal from the secondary effluent was significantly lower when lower TiO_2_ concentrations (0.5 g/L) were used, and even when a higher concentration of TiO_2_ (4 g/L) was used, the maximum removal of DOC and UV_254_ was only 23% and 52%, respectively, after 200 min of photocatalytic treatment. The reason proposed for the low removals compared to model HA systems is that the chemical structure of organic molecules present in the secondary effluent is less amenable to photocatalysis, and various salts contained in the wastewater can reduce the efficiency of oxidation via free radical scavenging.

The application of photocatalyst improved the permeate flux when higher TiO_2_ concentration was used. The flux improvement in a photocatalysis-ceramic membrane hybrid system was attributed to the formation of a more porous cake layer on the membrane covered with higher TiO_2_ particles when higher TiO_2_ concentration was used compared to that in lower TiO_2_ concentration. Furthermore, a positive effect of a photocatalyst on the permeate flux was observed after TiO_2_ adsorption and TiO_2_/UV treatment, as the filter cake had reduced organic compounds adsorbed between TiO_2_ particles in the filter cake.

LC-OCD analysis with online organic carbon detection revealed substances eluting in the biopolymer peak to be the main foulants in secondary effluent ceramic membrane filtration. While organic compound removal across the ceramic membrane after TiO_2_/UV photocatalytic treatment was low overall, the biopolymer fraction, as determined via LC-OCD, was removed to the greatest extent in accordance with its greater size. The hybrid system operated through non-selective hydroxyl radical oxidation and molecular separation, which progressively led to an effluent with lower biopolymer content that resulted in an increased proportion of the organic compounds being of LMW in the permeate. Substantial amounts of humic substances still remained after hybrid treatment. Monitoring the organic matter fractions with LC-OCD demonstrated that the reduction of effluent aromaticity (decreasing SUVA) was not strictly correlated with the complete depletion of humic substances in the effluents after hybrid treatment.

## Figures and Tables

**Table 1:** Characteristics of disinfected secondary effluent.

Parameters	Disinfected Secondary Effluent
pH	7.5–7.6
Conductivity (µS/cm)	1700–1800
Dissolved Organic Carbon (DOC) mg/L	9–10
Total Dissolved Solids (TDS) (mg/L)	1000–1100
UV 254 (cm^−1^)	0.16–0.17
True Colour (Pt-Co)	25–27
Turbidity (NTU)	1.9–2.0
Alkalinity (Bicarbonate Alkalinity (mg CaCO_3_/L))	120 *
Sulphate (mg/L)	94 *
Calcium (mg/L)	31 *
Magnesium (mg/L)	26 *
Potassium (mg/L)	27 *
Sodium (mg/L)	271 *
Chloride (mg/L)	420 *
Ammonia (mg/L)	0.2 *
Total Nitrogen (mg/)	11 *
Total Phosphorus (mg/L)	9 *

* Parameters were analysed in the Environmental division, ALS group laboratory.

**Table 2:** Removal of DOC and UV_254_ at various TiO_2_ concentrations after the hybrid system (TMP: 100 kPa, CFV: 0.4 m/s, UV intensity: 3.4 mW/cm^2^; pH: 7.5).

Experimental Conditions	After Photocatalysis		After Hybrid System	
	DOC Removal (%)	UV 254 Removal (%)	DOC Removal (%)	UV 254 Removal (%)
0.5 g/L TiO_2_	3	2	18	10
2 g/L TiO_2_	16	33	36	52
4 g/L TiO_2_	23	52	60	54
